# Supplementary material for: Enhanced Photodetection Range from Visible to Shortwave Infrared Light by ReSe2/MoTe2 van der Waals Heterostructure
Source: Nanomaterials (Basel). 2022 Aug 3;12(15):2664. doi: 10.3390/nano12152664 (PMC9370303; doi:10.3390/nano12152664)
Supplement: Supplementary file 1 [file nanomaterials-12-02664-s001.zip › nanomaterials-1780986-supplementary.pdf]

nanomaterials

Supplementary Materials for:

Enhanced Photodetection Range from Visible to Shortwave Infrared Light by $ReSe_2$/$MoTe_2$ van der Waals Heterostructure

Zhitao Lin [1,†], Wenbiao Zhu [2,†], Yonghong Zeng [2], Yiqing Shu [1], Haiguo Hu [2], Weicheng Chen [3] and Jianqing Li [1,*]

[1] School of computer science and engineering, Macau University of Science and Technology, Avenida Wai Long, Taipa, Macao 999078, China; 1809853gii30001@student.must.edu.mo (Z.L.); 1909853yii30001@student.must.edu.mo (Y.S.)

[2] Institute of Microscale Optoelectronics, Collaborative Innovation Centre for Optoelectronic Science & Technology, Key Laboratory of Optoelectronic Devices and Systems of Ministry of Education and Guangdong Province, College of Physics and Optoelectronic Engineering, Shenzhen Key Laboratory of Micro-Nano Photonic Information Technology, Guangdong Laboratory of Artificial Intelligence and Digital Economy (SZ), Shenzhen University, Shenzhen 518060, China; 1910454024@email.szu.edu.cn (W.Z.); zengyonghong777@163.com (Y.Z.); huhaiguo916@163.com (H.H.)

[3] Guangdong-HongKong-Macao Joint Laboratory for Intelligent Micro-Nano Optoelectronic Technology, Foshan University, Foshan 528225, China; chenwch@fosu.edu.cn

* Correspondence: jqli@must.edu.mo

† These authors contributed equally to this work.

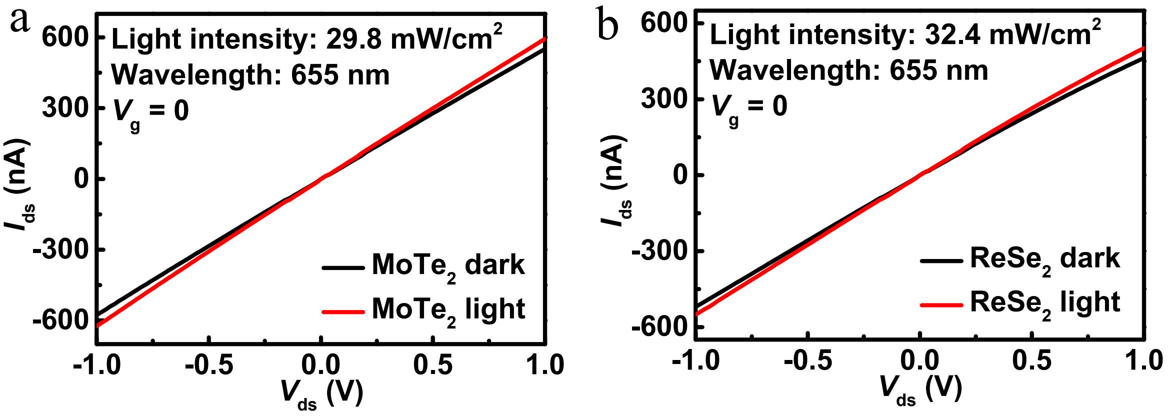

Figure S1. *I-V* characteristics of the individual (**a**) $ReSe_2$ nanosheets and (**b**) $MoTe_2$ nanosheets under dark and red light illumination.

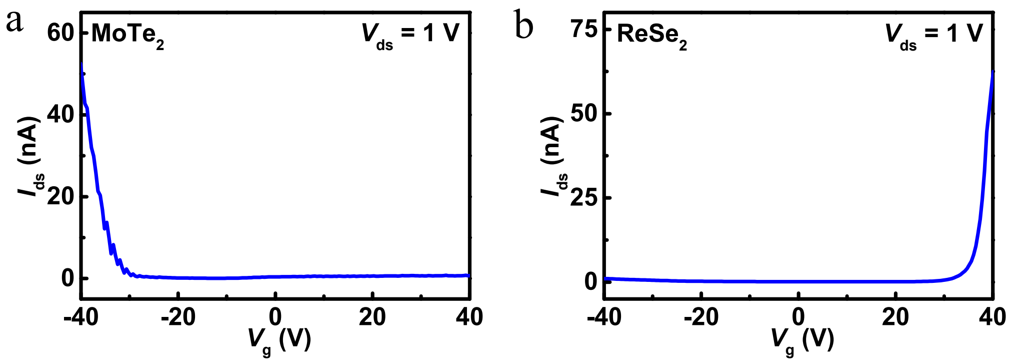

Figure S2. I_{ds}-V_g curves of the single $MoTe_2$ (**a**) and $ReSe_2$ (**b**) nanosheets FET.

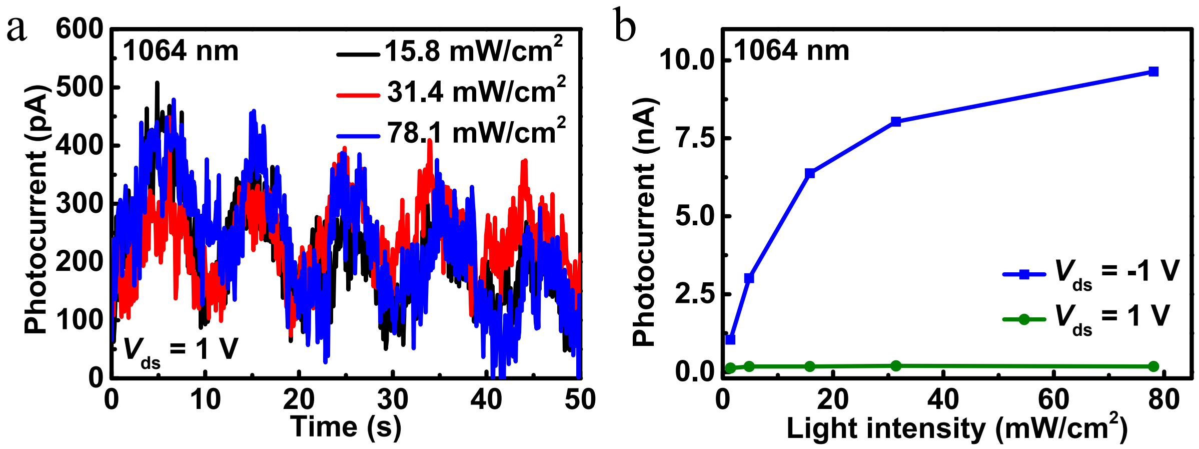

Figure S3. (**a**) Time-dependent photoresponse of the $ReSe_2/MoTe_2$ heterojunction under varied light intensities (1064 nm, V_{ds} =1 V), (**b**) The photocurrent as functions of light intensity under 1064 nm incident light (V_{ds}=±1 V).

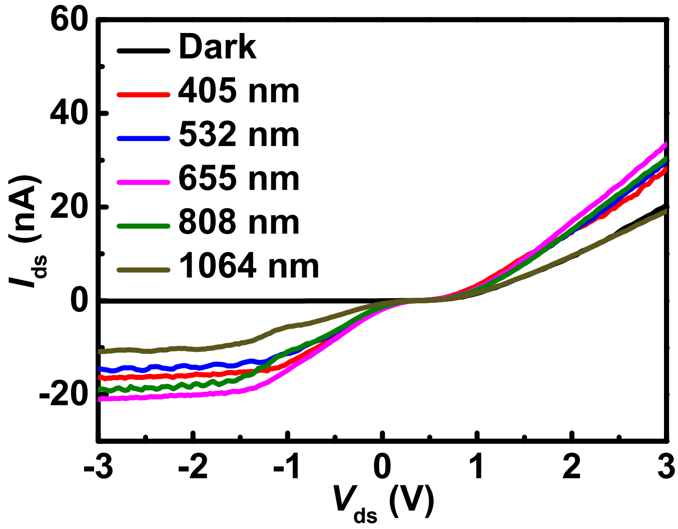

Figure S4. *I*–*V* curves of the photodetector under light illumination with different wavelengths.

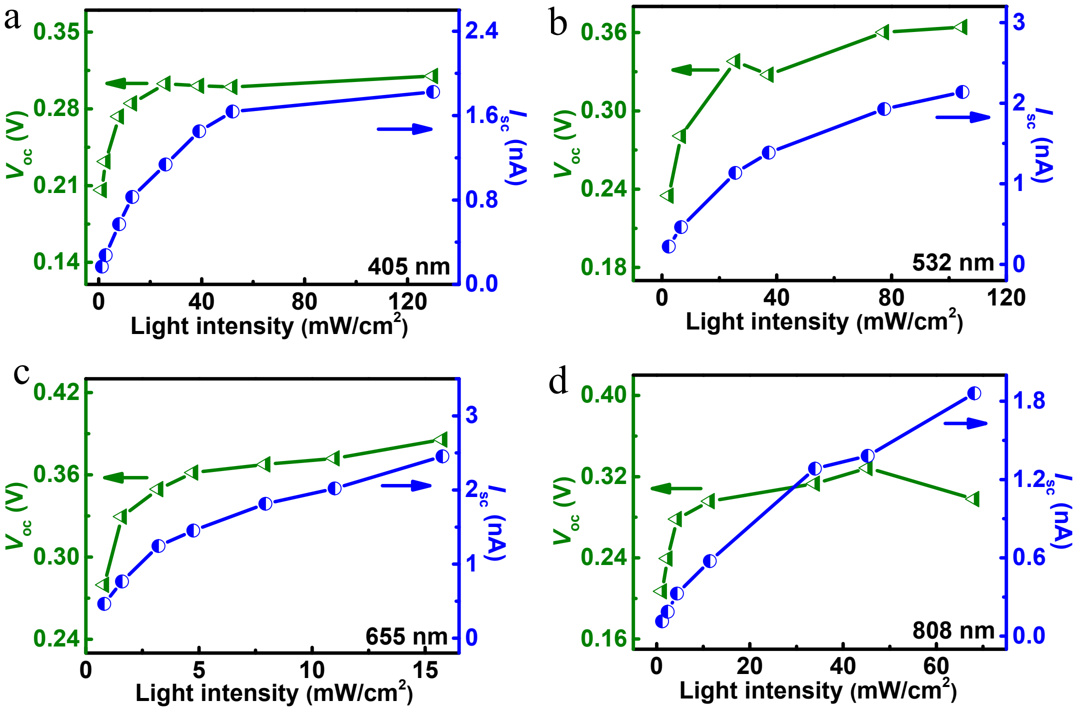

Figure S5. Extracted open-circuit voltage (V_{oc}, left axis) and short-circuit current (I_{sc}, right axis) as functions of light intensity under different wavelengths of 405 nm (**a**), 532 nm (**b**), 655 nm (**c**), and 808 nm (**d**).

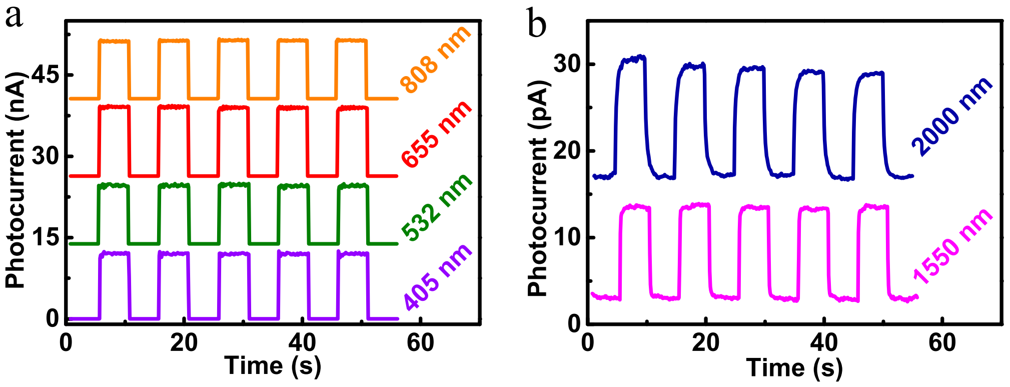

Figure S6. (**a–b**) Time-dependent photoresponse of the $ReSe_2/MoTe_2$ heterojunction under various wavelengths from visible to shortwave infrared (V_{ds}=-1 V and V_g=0 V). Measurements were carried out in the atmosphere at room temperature.

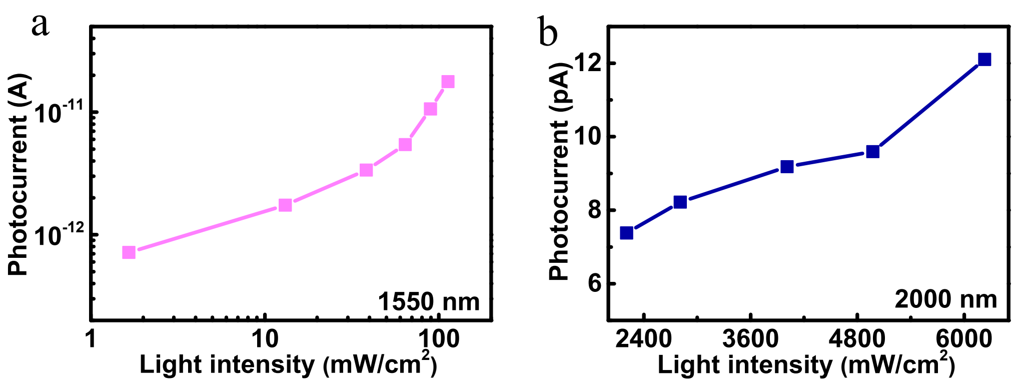

Figure S7. (**a**) Logarithmic plot of the photocurrent as functions of light intensity under 1550 nm incident light. (**b**) The photocurrent as functions of light intensity under 2000 nm incident light.

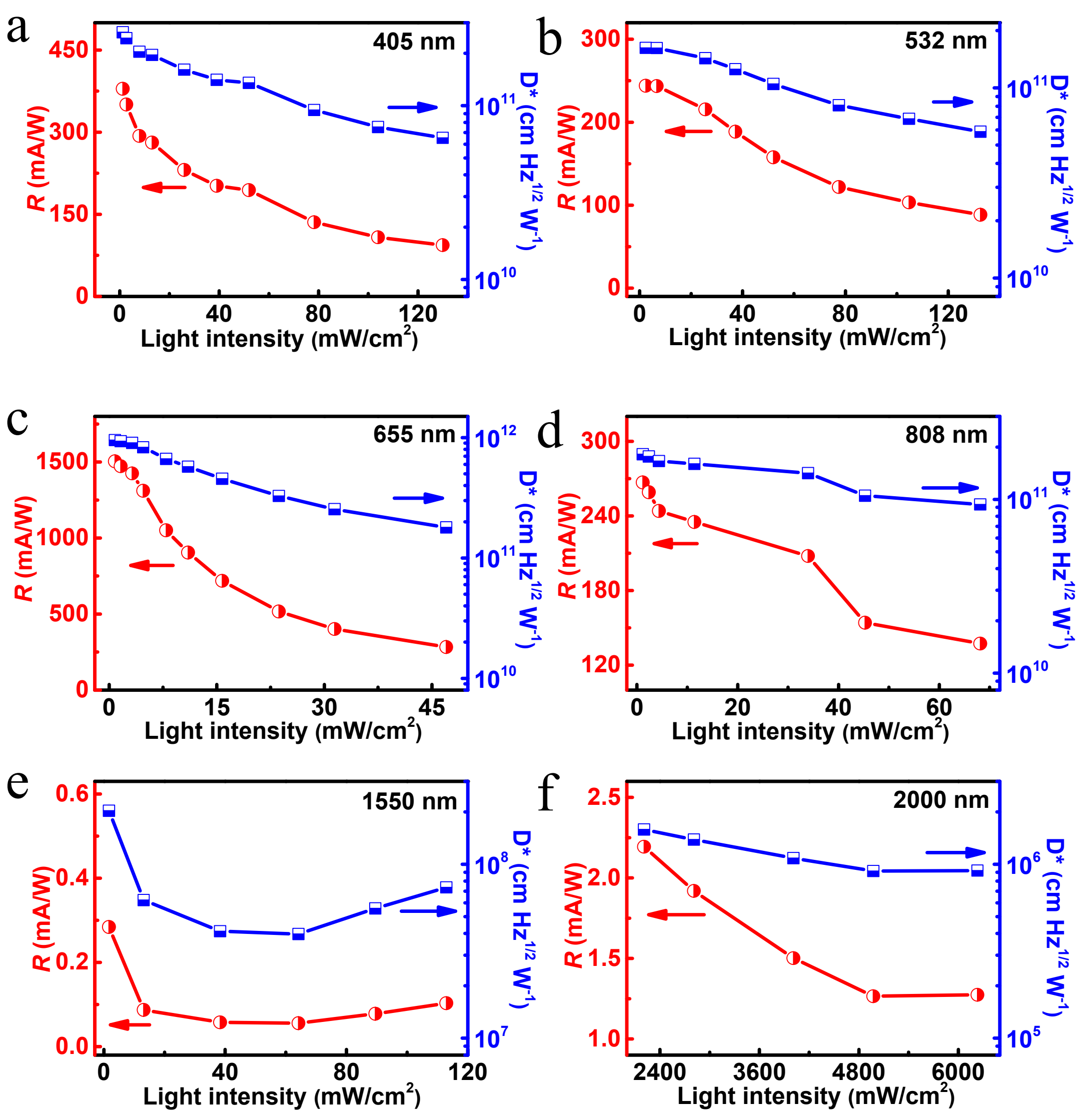

Figure S8. Responsivity and specific detectivity of the $ReSe_2/MoTe_2$ heterojunction photodetector as functions of light intensity under different wavelengths of (**a**) 405 nm, (**b**) 532 nm, (**c**) 655 nm, (**d**) 808 nm, (**e**) 1550 nm, (**f**) 2000 nm.

Table S1. Key parameter comparison list of $ReSe_2/MoTe_2$ heterojunction photodetector.

Structure	Thickness (nm)	Wavelength (nm)	Responsivity (A/W)	Detectivity (Jones)	Rise/Fall time (ms)	Ref
$ReSe_2/MoTe_2$	15.1/8.4	405-2000	1.05	6.66×10^{11}	5.6/4.2	This work
$ReS_2/ReSe_2$	64/48	400-550	2.1×10^{2}	NA	400	[1]
$ReSe_2/MoS_2$	60/7	633	6.75	NA	NA	[2]
$InSe/ReSe_2$	10.7/2.9	300-980	53.29	4.70×10^{11}	0.36/0.39	[3]
$WSe_2/ReSe_2$	6/50	405-980	0.57	NA	NA	[4]
$BP/ReSe_2$	5.9/6.5	405-633	0.44	NA	18.0/20.2	[5]
$BP/h\text{-}BN/ReSe_2$	5.8/0.91/10	532	0.012	28.9×10^{12}	NA	[6]
$GeSe/ReSe_2$	7/9	405-1550	2.89×10^{5}	4.91×10^{13}	0.016/ 0.02	[7]
$ReS_2/ReSe_2$	6.5/5.5	350-980	126.56	1.76×10^{11}	0.006/ 0.0089	[8]
$ReSe_2/WSe_2$	12/25	520	0.28	1.1×10^{12}	NA	[9]
$MoTe_2/ReSe_2$	5.5/5	455-980	5.6×10^{2}	8.1×10^{13}	NA	[10]
$Gr/ReSe_2/Gr$	14.5	220-1064	1.5×10^{7}	NA	5420/ 2980	[11]

References

1. Cho, A.-J.; Namgung, S. D.; Kim, H.; Kwon, J.-Y., Electric and photovoltaic characteristics of a multi-layer ReS2/ReSe2 heterostructure. *APL Materials* **2017,** *5* (7) , 076101.
2. Wang, X.; Huang, L.; Peng, Y.; Huo, N.; Wu, K.; Xia, C.; Wei, Z.; Tongay, S.; Li, J., Enhanced rectification, transport property and photocurrent generation of multilayer ReSe2/MoS2 p–n heterojunctions. *Nano Research* **2015,** *9* (2), 507-516.
3. Du, C.; Gao, H.; Du, W.; Li, J.; Leng, J.; Li, K.; Wang, W., High responsivity and broadband polarized photodetectors based on InSe/ReSe2 van der Waals heterostructures. *Journal of Alloys and Compounds* **2022,** 165586.
4. Ahn, J.; Ko, K.; Kyhm, J. H.; Ra, H. S.; Bae, H.; Hong, S.; Kim, D. Y.; Jang, J.; Kim, T. W.; Choi, S.; Kang, J. H.; Kwon, N.; Park, S.; Ju, B. K.; Poon, T. C.; Park, M. C.; Im, S.; Hwang, D. K., Near-Infrared Self-Powered Linearly Polarized Photodetection and Digital Incoherent Holography Using WSe2/ReSe2 van der Waals Heterostructure. *ACS Nano* **2021,** *15(11),* 17917-17925.
5. Shih, C.-C.; Huang, M.-H.; Wan, C.-K.; Jian, W.-B.; Kono, K.; Lin, Y.-F.; Ho, C.-H., Tuning Interface Barrier in 2D BP/ReSe2 Heterojunctions in Control of Optoelectronic Performances and Energy Conversion Efficiencies. *ACS Photonics* **2020,** *7* (10), 2886-2895.
6. Afzal, A. M.; Javed, Y.; Akhtar Shad, N.; Iqbal, M. Z.; Dastgeer, G.; Munir Sajid, M.; Mumtaz, S., Tunneling-based rectification and photoresponsivity in black phosphorus/hexagonal boron nitride/rhenium diselenide van der Waals heterojunction diode. *Nanoscale* **2020,** *12* (5), 3455-3468.
7. Afzal, A. M.; Iqbal, M. Z.; Dastgeer, G.; Nazir, G.; Eom, J., Ultrafast and Highly Stable Photodetectors Based on p-GeSe/n-ReSe2 Heterostructures. *ACS Appl Mater Interfaces* **2021,** *13* (40), 47882-47894.
8. Li, K.; Du, C.; Gao, H.; Yin, T.; Zheng, L.; Leng, J.; Wang, W., Ultrafast and Polarization-Sensitive ReS2/ReSe2 Heterostructure Photodetectors with Ambipolar Photoresponse. *ACS Appl Mater Interfaces* **2022**.
9. Tian, X.; Liu, Y., Van der Waals heterojunction ReSe2/WSe2 polarization-resolved photodetector. *Journal of Semiconductors* **2021,** *42* (3), 032001.
10. Jaffery, S. H. A.; Dastgeer, G.; Hussain, M.; Ali, A.; Hussain, S.; Ali, M.; Jung, J., Near-Direct Band Alignment of MoTe2/ReSe2 Type-II p-n Heterojunction for Efficient VNIR Photodetection. *Advanced Materials Technologies* **2022,** 2200026.
11. Elahi, E.; Khan, M. F.; Rehman, S.; Khalil, H. M. W.; Rehman, M. A.; Kim, D. K.; Kim, H.; Khan, K.; Shahzad, M.; Iqbal, M. W.; Basit, M. A.; Khan, F., Enhanced electrical and broad spectral (UV-Vis-NIR) photodetection in a Gr/ReSe2/Gr heterojunction. *Dalton Trans* **2020,** *49* (29), 10017-10027.
